# Vesiculobullous and Other Cutaneous Manifestations of COVID-19 Vaccines: a Scoping and Narrative Review

**DOI:** 10.1177/12034754231156561

**Published:** 2023-02-15

**Authors:** Farhan Mahmood, Janelle Cyr, Amy Li, Jennifer Lipson, Melanie Pratt, Jennifer Beecker

**Affiliations:** 1 12365 6363 Department of Medicine, University of Ottawa, Ottawa, ON, Canada; 2 153006 Division of Dermatology, The Ottawa Hospital, Ottawa, ON, Canada

**Keywords:** COVID-19, vaccine reaction, vesiculobullous reaction, bullous, cutaneous manifestations

## Abstract

As coronavirus disease (COVID-19) vaccines continue to be administered, dermatologists play a critical role in recognizing and treating the cutaneous manifestations (CM) associated with the vaccines. Adverse cutaneous reactions of COVID-19 vaccines reported in the literature range from common urticarial to rare vesiculobullous reactions. In this study, we performed a (1) scoping review to assess the occurrences of vesicular, papulovesicular, and bullous CMs of COVID-19 vaccines and their respective treatments, and (2) a narrative review discussing other common and uncommon CMs of COVID-19 vaccines. Thirty-six articles were included in the scoping review, and 66 articles in the narrative review. We found that vesicular, papulovesicular, and bullous lesions are infrequent, reported mostly after the first dose of Moderna or Pfizer vaccines. Eleven of the 36 studies reported vesicular reactions consistent with activation or reactivation of the herpes zoster virus. Most vesicular and bullous lesions were self-limited or treated with topical corticosteroids. Other CMs included injection-site, urticarial or morbilliform reactions, vasculitis, toxic epidermal necrolysis, and flaring of or new-onset skin diseases such as psoriasis. Treatments for CMs included topical or oral corticosteroids, antihistamines, or no treatment in self-limited cases. Although most CMs are benign and treatable, the data on the effect of systemic corticosteroids and immunosuppressive therapies on the immunogenicity of COVID-19 vaccines is limited. Some studies report reduced immunogenicity of the vaccines after high-dose corticosteroids use. Physicians may consult local guidelines where available when recommending COVID-19 vaccines to immunosuppressed patients, and when using corticosteroids to manage the CMs of COVID-19 vaccines.

## Introduction

Since the start of the coronavirus disease (COVID-19) pandemic, dermatologists have played an important role in the diagnosis and management of the varied and now well-described cutaneous manifestations of the Severe Acute Respiratory Syndrome Coronavirus 2 (SARS-CoV-2). As vaccines became rapidly administered across the globe, dermatologists have also been integrally involved in recognizing and treating the cutaneous manifestations of COVID-19 vaccines. While the vaccines available worldwide are considered safe and effective, there are reports of cutaneous reactions after the first and subsequent doses of several vaccines.^1^ Some common vaccines approved globally include mRNA-based and viral vector-based vaccines such as Moderna Spikevax^TM^ (mRNA-1273; referred to as Moderna), Pfizer-BioNTech Comirnaty^TM^ (BNT162b2; referred to as Pfizer), AstraZeneca Vaxzevria^TM^ (AZD1222 and ChAdOx1-S [recombinant]; referred to as AstraZeneca), and Janssen (Johnson & Johnson^TM^; JNJ-78436735; referred to as Janssen),^2^ Sinovac-Coroavac^TM^ (referred to as Sinovac),^3^ and COVAXIN^®^ (Bharat Biotech BBV152; referred to as Covaxin).^4^

The cutaneous manifestations of the SARS-CoV-2 virus have been reported and summarized extensively in the literature and can be categorized based on their patho-mechanisms into viral exanthems and cutaneous eruptions secondary to systemic consequences.^5^ A meta-analysis of over 2000 articles found that the prevalence of cutaneous manifestations in COVID-19 was 5.69%.^6^ Reported cutaneous manifestations include morbilliform, pseudo-chilblain, pernio-like, urticarial, macular erythema, vesicular, papulosquamous, retiform purpura, livedo, and necrosis, with varying frequencies, which are presented in Supplementary Table 1.^5-10^

While the current knowledge of cutaneous manifestations of SARS-CoV-2 has been reviewed in the literature, data on adverse cutaneous reactions to SARS-CoV-2 vaccines have mostly been reported in case reports or series and/or registry-based retrospective reviews. Cutaneous manifestations are also being reported through the Vaccine Adverse Event Reporting System^11^ and the American Academy of Dermatology COVID-19 Registry.^1^ McMahon et al. have reported several common cutaneous reactions including injection site, urticarial, and morbilliform reactions.^12^ A few studies have contrastingly reported uncommon cutaneous manifestations including vesicular, papulovesicular, and bullous-like reactions.^13^

The primary objective of the scoping review is to review the vesicular, papulovesicular, and bullous cutaneous manifestations of COVID-19 vaccines reported in the literature and their frequencies after the first and subsequent doses, and the modalities of treatments utilized. The secondary objective of this paper is to conduct a narrative review of other common and uncommon cutaneous manifestations while highlighting the clinically salient treatment options for Dermatologists and physicians.

## Methods

Two concurrent reviews were performed to fulfill the aim of this paper. First, a scoping^14^ review was conducted in accordance with the AMSTAR 2 (A MeaSurement Tool to Assess systematic Reviews) reporting guidelines^15^ which included patients who received either the first or subsequent doses of COVID-19 vaccines with reported vesicular, papulovesicular, or bullous cutaneous manifestations. A second broader narrative review discussion included articles reporting on all other common and uncommon cutaneous manifestations of COVID-19 vaccines.

### Eligibility Criteria

Due to the limited number of randomized controlled trials (RCTs) and controlled clinical trials (CCTs) reporting the cutaneous manifestations of COVID-19 vaccines, all peer-reviewed article types were included in both the scoping and narrative reviews. These included case reports, case series, observational, retrospective, registry-based retrospective reviews, RCTs, and CCTs. Resultantly, we were unable to conduct a quality assessment of the studies included in the review due to the high level of heterogeneity and the inclusion of non-interventional and case report studies. For both the scoping and narrative reviews, the study subjects must have received the COVID-19 vaccine. There were no restrictions on the type of vaccine in the scoping review; however, the narrative review discussion was focused on the vaccines approved by Health Canada (Moderna, Pfizer, AstraZeneca, and Janssen).^2^ No date restrictions were applied and only studies reported in English were included in both reviews. Studies were excluded from the narrative review if (1) they did not report the cutaneous manifestations after the first or subsequent vaccine doses, (2) they did not clearly define or describe the cutaneous manifestation and its onset, or if (3) the studies did not include vaccines approved by Health Canada.^2^

During the full-text screening process, articles with a focus on cutaneous manifestations of COVID-19 vaccines were retrieved for the narrative review. These included registry studies, retrospective reviews, case reports, and case series. Due to the broad spectrum of common cutaneous manifestations reported in the literature, we excluded articles that did not discuss vaccines approved by Health Canada.

### Outcomes

The following outcomes were assessed:

1. Type of cutaneous reaction
2. Frequency of cases reported after each dose
3. Time to onset after the vaccine
4. Duration of cutaneous reaction
5. Treatment of the reaction

### Electronic Searches

The search was conducted using EMBASE, Web of Science, and PubMed from their inception to the present on January 18th, 2022. Articles were screened to identify additional references. The following search terms were used: vesiculopapular, papulovesicular, papular, vesicular, bullae, bullous, cutaneous reactions, skin reactions, dermatology reactions, cutaneous side effects, adverse reactions, and vaccination, vaccine, Pfizer-BioNTech Comirnaty, Moderna Spikevax, Pfizer-BioNTech, mRNA-1273, Moderna, Pfizer, and SARS-CoV-2, coronavirus, Covid-19, Covid.

### Data Collection and Extraction

Titles, abstracts, and full-text articles were dual-screened by two reviewers (F.M. and A.L.) on the Covidence platform.^16^ Data were extracted by three reviewers (F.M., A.L., J.C.). Data were presented via tables and described qualitatively. Due to the heterogeneity of the study types, populations, and vaccines administered a combined analysis of the data or meta-analysis could not be performed.

## Results

The scoping review yielded 1984 articles, of which 793 were duplicates (Supplementary Figure 1). For the scoping review, the titles and abstracts of 1191 articles were screened, and 203 articles were included in the full-text review. Of these, a total of 36 articles were included in the scoping review. The reason for exclusion included no discussion of vesicular, papulovesicular, or bullous reactions (*n* = 167). Twenty-seven^12,13,17-41^ of the 36 articles described nonspecific vesicular, papulovesicular, or bullous reactions, compared to eleven^18,24,42-50^ discussing vesicular eruptions in concordance with new-onset or reactivation of the herpes zoster virus. During the screening process, a total of 66 articles discussing other cutaneous manifestations were retrieved from the full-text screening to extract data in tabular formats and were discussed in our narrative review.

### Vesicular and Papulovesicular Reactions

Vesicular and papulovesicular reactions have been reported after the Moderna, Pfizer, AstraZeneca, and Sinovac vaccines (Supplementary Table 2). Four studies including RCTs, and case series reported vesicular or papulovesicular reactions after the first Moderna vaccine and three after the second dose.^12,17-20^ Cases of vesicular reactions ranged from <0.1% (15185 total first doses) to 4.8% (147 total first doses) after the first dose, compared to 1% (102 total second doses) to 5% (40 total second doses) after the second dose. The time to onset after the first and second doses ranged from 6.4 to 28 days and 3-48 days, respectively. Most of these reactions spontaneously resolved around 7 days.

Six studies similarly reported vesicular and papulovesicular reactions after the first dose of Pfizer compared to four reporting it after the second dose.^12,18,20-23^ Freeman et al. reported up to 9.6% (114 total first doses) and 10% (140 total second doses) of cases after the first and second doses of Pfizer, respectively.^18^ Vaccaro et al. reported a case series of 8 patients who developed vesicular rashes after the first Pfizer or AstraZeneca doses combined, occurring around 4-12 hours after the vaccines.^23^ The reactions persisted for up to 14 days. Tammaro et al. similarly reported two cases of vesicular reactions after the second Pfizer dose occurring in 64 and 56-year-old females.^22^ The reactions consisted of round erythematous, painful, and pruritic nodules and vesicles.

Cases of vesicular reactions have also been reported after the first dose of the AstraZeneca vaccine in 4 studies.^20,24-26^ Up to 8.4% (95 total first dose AstraZeneca) cases were reported after the first AstraZeneca vaccine, and these reactions generally persisted for 3 days. Rerknimitr et al. also reported vesicular lesions after both the first and second doses of Sinovac.^24^

### Bullous Reactions

Most of the bullous reactions from COVID-19 vaccines were reported through case reports and case series (Supplementary Table 2). Five studies reported bullous reactions after the first and second doses of Moderna.^13,18,27-30^ Amongst the patients receiving the Moderna vaccine, 3.1% of 447 patients developed bullous eruptions after the first Moderna dose compared to 2.6% of 223 patients after the second dose. Two cases had worsened bullous eruptions after the second Moderna dose. Most of the bullous reactions resolved within 2-3 weeks. The onset of eruptions after the vaccines averaged around 9.9 and 5.6 days after the first and second doses of Moderna respectively.

Bullous reactions after the Pfizer vaccine have mostly been described in 13 studies including 12 case reports or case series and 1 registry study, 4 of which were confirmed cases of bullous pemphigoid.^13,18,23,27,28,31-38^ Upon combining the patients receiving the Pfizer vaccines, 13.5% of 178 cases developed bullous eruptions after the first dose reported in 12 studies compared to 13.5% of 156 cases after the second dose reported in 8 studies. Four cases had reactions after both the first and second vaccines and 2 cases had ongoing or unresolved lesions when receiving the second dose, which persisted after the second dose as well. Juay et al. reported 3 cases including dyshidrotic eczema, acute generalized pustulosis, and bullous pemphigoid.^31^ The average time to onset after the first and second dose was 8.5 and 3.95 days, respectively. Most of these lesions resolved by 3 weeks after the vaccine doses with 4 cases ongoing at 6 weeks or more.

Vaccaro et al. reported 2 cases of vesicular lesions coalescing into a single bulla after the AstraZeneca or Pfizer vaccines.^23^ This study did not specify whether the Pfizer or AstraZeneca vaccines caused the bullous reaction; however, Vaccaro et al. do note that 12 injection‐site skin reactions were described after the Pfizer vaccine and 16 after the AstraZeneca vaccine out of a total sample size of 16217 vaccinated with Pfizer and 1377 vaccinated with AstraZeneca.^23^ Three other case reports describe bullous reactions after the first (2 cases) and second (1 case) doses of Astrazeneca.^39-41^ Biopsy findings from 2 of the case reports from AstraZeneca vaccine reactions demonstrated linear IgA bullous dermatosis and a generalized bullous fixed drug eruption on the abdomen, trunk thighs, and extremities.^40,41^

### Varicella-Zoster Virus (VZV) Reactions

Five^18,24,42-44^ studies have reported new-onset or activation of VZV after Pfizer, AstraZeneca, or Sinovac vaccines, which have presented as blisters and vesicles with maculopapular rashes (Supplementary Table 3).^18,24,42-53^ Most reactions occurred after the first dose, presenting 4.6 days after the vaccine and lasting for up to 2 weeks. Six studies^45-50^ reported reactivation of VZV in patients with previous varicella cutaneous eruptions presenting after 9.8 and 6.8 days after the first and second doses of COVID-19 vaccines, respectively. Reactivations were most commonly associated with the second dose of the Pfizer vaccine. VZV reactions were mostly treated with antiviral therapies.

### Treatment of Vesicular, Papulovesicular, and Bullous-Like Reactions

Most vesicular or papulovesicular eruptions were self-limited and resolved within 7 days. Reported outpatient treatments included topical corticosteroids and antihistamines. Only one case required hospitalization for generalized bullous erythema multiforme; however, the authors do not specify the management and outcome of this patient.^19^ One case of herpes zoster reactivation after Pfizer initially did not respond to 40 mg of prednisone, 25 mg of hydroxyzine, and 2% mupirocin ointment but later self-regressed (Supplementary Tables 2 and 3).^47^

The majority of bullous reactions were managed with oral prednisone, topical corticosteroids, and emollients. Combinations of topical or oral steroids and mycophenolate mofetil or doxycycline, niacinamide, and antihistamines were also reported with success. A case of dyshidrotic eczema with bullae was treated with topical betamethasone dipropionate ointment.^31^

## Narrative Review of Common and Uncommon Cutaneous Manifestations

In addition to vesicular, papulovesicular, and bullous reactions, several other common and uncommon cutaneous manifestations have been reported. Uncommon manifestations included those reported merely in case reports or small case series and/or reported in less than 5 articles we screened.

### Common Cutaneous Manifestations

Common cutaneous manifestations are summarized in Supplementary Table 4.^12,17-24,27,43,44,54-77^ These included delayed large local arm reactions, local injection site reactions, urticaria or urticarial reactions, morbilliform or maculopapular eruptions, pityriasis rosea (PR)-like eruptions, pernio or chilblains, dermatitis, cutaneous small vessel vasculitis (CSVV), ecchymosis, petechiae, purpura, and lymphadenopathy. The reactions were mainly reported with Moderna and Pfizer and occasionally with AstraZeneca, Sinovac, and Covaxin.

Many of these common reactions were reported through randomized controlled trials, registry studies, case series, or observational studies. McMahon et al. reported a registry-based study that gathered information about cutaneous manifestations following Pfizer and Moderna COVID-19 vaccinations.^12^ They found that delayed large local reactions were the most common, with pernio/chilblains and PR-like reactions being less common.

Most urticarial reactions from Moderna occurred around 2-28 days after the first dose and lasted around 2-30 days. From the second dose, urticarial reactions occurred around 2 days after the vaccination and lasted around 2-5 days. Urticarial reactions from Pfizer occurred around 20 minutes to 10 days after the first dose, lasting around 12 hr up to 28 days, and occurred from 30 minutes to 5 days after the second dose, lasting around 12 hr up to 14 days (Supplementary Table 4).

Morbilliform or maculopapular eruptions were reported to be caused by Moderna, Pfizer, AstraZeneca, and Sinovac. The reactions occurred around 3-28 days after the first dose of Moderna, lasting 4-10 days, and occurred around 1-2 days after the second dose, lasting from around 2.5-35 days. The reaction occurred around 1-7.5 days after the first dose of Pfizer, lasting around 4-12 days, and occurred around 4 hr to 2 days after the second dose, lasting around 2.5 days. There were a handful of morbilliform or maculopapular reactions after the AstraZeneca vaccination, mostly after the first dose (Supplementary Table 4).

Dermatitis was also reported after Moderna and Pfizer vaccination occurring around 5 days after the first and second doses of Moderna. With Pfizer, the reaction occurred 2.5-21 days after the first dose, lasting up to several weeks, and occurred 4-12 days after the second dose, lasting 8-21 days.

### Flares of Pre-Existing Dermatoses

Flares of pre-existing psoriasis were also reported, predominantly after the Pfizer vaccine,^21,51-53^ in addition to cases of vasculitis (Supplementary Table 3). One case of worsening psoriasis was reported after the first Moderna dose compared to 6 cases after the second dose.^51^ After the Pfizer vaccine, 2 cases of either pustular palmoplantar psoriasis or de novo nail psoriasis were reported after both first and second doses.^52,53^ Wei et al. further presented 57 flares of psoriasis after the first doses of Pfizer, Moderna, and Janssen vaccines combined compared to 19 flares reported after the second dose, 5 of which recurred after the first and second doses of Moderna or Pfizer (Supplementary Table 3).^51^

### Uncommon Cutaneous Manifestations

Several cases of uncommon cutaneous manifestations of vaccines have been reported and are summarized in Supplementary Table 5.^18,21,24,43,51,68,78-105^ These include toxic epidermal necrolysis (TEN), Stevens-Johnson syndrome (SJS), skin necrosis, angioedema, edematous infiltrated plaques, Sweet syndrome, acute generalized exanthematous pustulosis, erythema multiforme (EM), erythema nodosum (EN), radiation recall dermatitis, papular acrodermatitis, cutaneous lichen planus (LP), new-onset and exacerbation of cutaneous lupus erythematosus (cLE), T-cell predominant cutaneous lymphoid hyperplasia, lichenoid reactions, pityriasis lichenoides et varioliformis Acuta (PLEVA), molluscum contagiosum, cutaneous mucormycosis at the injection site, livedo racemosa, fixed drug eruption (FDE), vulvar aphthae, purpura due to Evan’s syndrome, and dermatomyositis. New-onset psoriasis and vitiligo have also been reported. These reactions were reported after the first doses of the Pfizer vaccine followed by Moderna then AstraZeneca. It is unclear whether this is because more people have received their first dose than subsequent doses. Five reactions initiated after the first dose recurred or exacerbated after the second dose including vitiligo, cLE, PLEVA, and angioedema. Five patients were advised not to receive the second dose of the vaccine after reacting to the first dose or chose not to themselves. The time to onset for all uncommon reactions was around 7.5 days after the administration of the vaccines.

Wei et al. reported one case of new-onset psoriasis after the second dose of Moderna in a case series, and 22 cases (6 guttate type psoriasis) after the first dose of Pfizer, Moderna, and Janssen in a retrospective review of the CDC VAERS (Centre for Disease Control and Prevention - The Vaccine Adverse Event Reporting System) reports from December 2020 to August 2021.^51^ Freeman et al. have also reported several new-onset dermatoses after the first and second doses of Moderna and Pfizer including lichen planus, psoriasis, and morphea.^18^

Reactions to previously placed hyaluronic acid soft tissue fillers have been reported after Pfizer’s first and second doses (3 cases) and after the second dose of Moderna (1 case). Most of the reactions were self-limited resolving within 1 week, with 2 cases requiring either methylprednisolone or removal of the filler product (Supplementary Table 3).^103-105^

### Treatment of Non-Vesiculobullous Reactions

Reported treatments for common cutaneous manifestations included topical corticosteroids, oral corticosteroids taper such as prednisolone or prednisone, antihistamines, or no treatment in self-limiting cases. Vaccine-induced psoriasis was reported to improve with topical steroids, apremilast, phototherapy, and biologics including risankizumab and tildrakizumab. Most reactions improved with treatment (Supplementary Table 4). Reactions that did not improve included an urticarial reaction after Moderna treated with topical mometasone,^62^ a case of acute generalized exanthematous pustulosis managed with diphenhydramine and topical hydrocortisone cream,^84^ and a case of cLE after Pfizer which initially did not respond to oral prednisolone 10 mg but responded to pulse therapy with 60 mg of prednisolone tapered over 3 weeks with topical mometasone ointment.^93^ Cases of common or uncommon manifestations which were hospitalized included PR-like eruptions, CSVV, lichenoid reactions, TEN, and SJS (Supplementary Table 5).

## Discussion of Treatment Considerations and Guidance

Corticosteroids are most commonly reported as the management of cutaneous COVID-19 vaccine reactions; however, the immunosuppressive impacts of systemic corticosteroids on the immunogenic response to vaccination should be considered prior to treatment.^106,107^ Recent studies have assessed the immunogenicity of COVID-19 vaccines in immunocompromised patients; however, data is limited on patients receiving corticosteroids for a cutaneous manifestation of the COVID-19 vaccine. The 2021 American College of Rheumatology (ACR) guidelines^108^ suggested that conventional and targeted immunomodulatory or immunosuppressive medications should be held for one to 2 weeks (as disease activity allows) after each COVID-19 vaccine dose. There was no consensus as to whether a vaccine response might be blunted in prednisone doses of more than 20 mg/day. Deepak et al. showed that corticosteroids independent of their doses had a 10-fold reduction in humoral responses to COVID-19 vaccines when compared to the immunocompetent controls.^109^ However, Yang et al. found that short-term use of low-dose corticosteroids (median total of 30 mg prednisolone equivalent) did not hinder the vaccine’s immunogenicity but rather reduced the vaccine’s reactogenicity.^110^ Reactogenicity is defined as the local and systemic complications of the vaccine.

It has previously been reported that doses of up to 20 mg/day of prednisone or equivalents do not suppress responses to inactivated vaccines, as these doses are not considered immunosuppressive.^106,111^ Doses of 20 mg/day or more of corticosteroids for 2 or more weeks or more than 40 mg for more than 1 week are considered immunosuppressive.^106,107^

Other immunosuppressive agents including B-cell depletion agents also reduced the immunogenicity of COVID-19 vaccines up to 36-fold, especially when vaccinated within 6 months of B-cell depleting therapy.^109^ JAK inhibitors and antimetabolites including methotrexate also blunted antibody titers in a multivariate regression analysis. TNF inhibitors, IL-12/23 inhibitors, and integrin inhibitors had a modest impact on antibody formation and neutralization. Notably, patients infected with COVID-19 receiving B-cell depletion therapies have increased mortality compared to patients on methotrexate. Thus, B-cell depleting agents may need to be held or additional COVID-19 vaccines may be required.^109^ Furthermore, although evidence is limited, current guidelines suggest continuing biologics, small molecules, or antimetabolites in inflammatory bowel disease and psoriasis patients when administering the COVID-19 vaccine. Certain immunosuppressants including methotrexate and JAK inhibitors should be ideally held for 1 week after each COVID-19 vaccine dose for patients with well-controlled diseases.^109^ Current guidelines in inflammatory bowel disease and psoriasis do not suggest holding biologics, small molecules, or antimetabolites prior to vaccination against COVID-19; however, this could be decided on a case-by-case basis considering both the half-life of the drug and the clinical disease activity.^112,113^

Other immunocompromised groups include organ transplant recipients and patients with cancers. Previous studies showed that only 47.5% of liver transplant recipients had positive antibodies against COVID-19 after the Pfizer vaccine compared to 100% of the control group. Predictors for the negative response included treatment with high-dose prednisone in the last 12 months.^114^ A systematic review and meta-analysis similarly found that seroconversion after the first dose of mRNA or non-mRNA-based vaccines was half as likely in patients with hematological cancers, immune-mediated inflammatory disorders, and solid cancers, and 16 times less likely in organ transplant recipients. The seroconversion after subsequent second and third doses of the vaccine improved across all immunocompromised patients.^115^ Thus, it was concluded that targeted interventions for immunocompromised patients, including a third booster dose of COVID-19 vaccines should be administered.

## Limitations and Next Steps

This scoping review is limited to papulovesicular and vesiculobullous cutaneous manifestations of COVID-19 vaccines. Due to limited studies reporting such manifestations, we included all studies we identified and did not score for quality. As such, there were large variations in sample sizes and heterogeneity in how the outcomes were reported; thus, data synthesis was difficult. While we discuss other cutaneous manifestations in a narrative review style, these studies were identified from our focused search strategy for the scoping review. We did not include all studies reporting all cutaneous manifestations of COVID-19; however, given the broad nature of this topic, a scoping or systematic review on all cutaneous manifestations of COVID-19 vaccines would be unfeasible. Given many studies did not provide patient’s comorbidities or medication histories and we did not record these outcomes, it is difficult to determine whether some reported cutaneous manifestations are truly secondary to COVID-19 vaccines or developed concurrently, spontaneously or due to other comorbidities or drug reactions. Future studies may control for population, vaccine, and temporal variations by conducting prospective and/or retrospective studies assessing cutaneous manifestations in a large relatively healthy population receiving the same vaccine around the same time. Ideally, all patients would be followed at specific time points. The pathophysiology underlying several cutaneous manifestations should also be elucidated.

## Conclusion

In this review, we explored cutaneous manifestations of COVID-19 vaccines with a particular focus on vesicular, papulovesicular, and bullous lesions. Bullous and papulovesicular reactions are uncommon cutaneous manifestations of the COVID-19 vaccines, reported in a total of 36 case reports or case series occurring more often after the first dose of Moderna or Pfizer vaccines. Eleven of these 36 studies reported activation or reactivation of the herpes zoster virus after the COVID-19 vaccines, which presented with vesicular and papulovesicular lesions. Most of these lesions were self-limited or were treated with topical corticosteroids alone.

Across 66 studies, we found a total of 12 common CM and 17 uncommon CM. Common CM included local arm reactions, urticaria or urticarial reactions, morbilliform eruptions, PR-like eruptions, pernio or chilblains, dermatitis, leukocytoclastic vasculitis, ecchymosis, petechiae, purpura, and lymphadenopathy. Uncommon CM included TEN, SJS, skin necrosis, angioedema, infiltrated plaques, Sweet’s syndrome, acute generalized exanthematous pustulosis, EM, EN, radiation recall dermatitis, papular acrodermatitis, LP, new-onset and exacerbation of cutaneous lupus erythematosus, T-cell predominant cutaneous lymphoid hyperplasia, lichenoid reactions, PLEVA, molluscum contagiosum, cutaneous mucormycosis, livedo racemosa, FDE, vulvar aphthae, purpura due to Evan’s syndrome, and dermatomyositis. The reactions were most commonly associated with the first doses of Moderna and Pfizer vaccines, most of which were self-limited and managed with oral or topical corticosteroids. By reviewing the cutaneous manifestations associated with COVID-19 vaccines, we aim to help physicians recognize and manage patients presenting with manifestations.

## Supplemental Material

Table S1 - Supplemental material for Vesiculobullous and Other Cutaneous Manifestations of COVID-19 Vaccines: a Scoping and Narrative Review Click here for additional data file. Supplemental material, Table S1, for Vesiculobullous and Other Cutaneous Manifestations of COVID-19 Vaccines: a Scoping and Narrative Review by Farhan Mahmood, Janelle Cyr, Amy Li, Jennifer Lipson, Melanie Pratt and Jennifer Beecker in Journal of Cutaneous Medicine and Surgery
