# Supplementary material for: Vesiculobullous and Other Cutaneous Manifestations of COVID-19 Vaccines: a Scoping and Narrative Review
Source: J Cutan Med Surg. 2023 Feb 15;27(3):260–70. doi: 10.1177/12034754231156561 (PMC10291118; doi:10.1177/12034754231156561)
Supplement: Table S1 - Supplemental material for Vesiculobullous and Other Cutaneous Manifestations of COVID-19 Vaccines: a Scoping and Narrative Review [file sj-pdf-1-cms-10.1177_12034754231156561.pdf]

Supplementary File

Figures.

Supplementary Figure 1. Overview of Studies Identified and Included in the Scoping Review

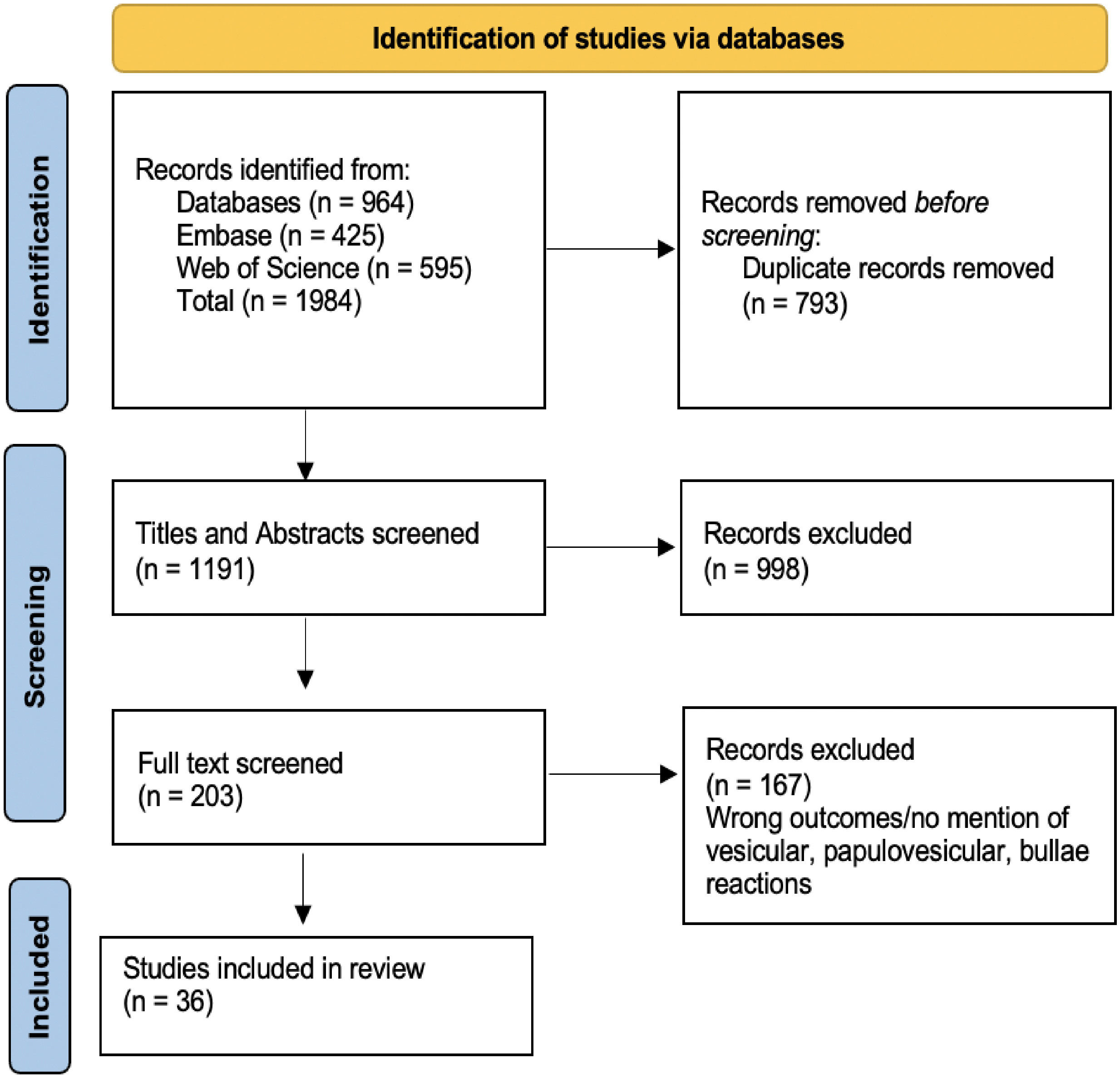

Tables.

Supplementary Table 1. Commonly Reported Cutaneous Manifestations of SARS-CoV-2 infection

Cutaneous Manifestation	Reported Incidence/Occurrence (%)
Pernio-like	18 - 63
Maculopapular/morbilliform	20.5 - 47
Papulovesicular	7.8 - 29.4
Urticarial	16 - 20.5
Pseudo-chilblain	7.8 - 19
Macular erythema	13
Papulosquamous	4 - 9.9
Purpura	0.6 - 6.4
Ecchymosis	5
Acral necrosis	2.2
Livedo racemosa	0.6
References: 5-10	

Supplementary Table 2. Studies Reporting Vesicular, Papulovesicular, and Bullous Reactions after the COVID-19 Vaccinations

**Study (Author, year)**	**Vaccine Type**	**Type of study**	**% Of cases after 1st dose (n= total sample size for the vaccine type)**	**Time to onset after vaccine 1 (mean days)**	**Duration of reaction (mean days)**	**Did reaction recur with second dose**	**% Of cases after 2nd dose (n= total sample size for the vaccine type)**	**Time to onset after vaccine 2 (mean days)**	**Duration of reaction (mean days)**	**Treatment (if noted)**	**Additional Comments**
Vesicular or Papulovesicular Reactions											
McMahon, 2021	Moderna	Randomized controlled trial	1.5 (267)	7	7	NR	1 (102)	3	7		
Baden, 2021	Moderna	Randomized controlled trial	<0.1 (15185)	28		NR					
Freeman, 2022	Moderna	Registry Study	3.3 (427)			NR	3.7 (214)				
Burlando, 2022	Moderna	Case series				NR	100 (1)	48		Hospitalized	-Patient required hospitalization for generalized bullous erythema multiforme, which occurred 48 hours after the second vaccine dose
Català, 2022	Moderna	Cross-sectional study	4.8 (147)	6.4	19.3	NR					
McMahon, 2021	Pfizer	Randomized controlled trial	8.8 (34)	7	7	NR	5 (40)	3	7		
Freeman, 2022	Pfizer	Registry Study	9.6 (114)			NR	10 (140)				
Niebel, 2021	Pfizer	Retrospective analysis/case series				NR	15.4 (13)	5		Topical corticosteroid<br>Topical Fucidin	-Papulovesicular/vesicular reaction
Tammaro, 2021	Pfizer	Case series				NR	100 (2)	1	7	Topical corticosteroid	-64-year-old female with round indented painful, erythematous, pruritic nodule at injection site<br>-56-year-old female with erythematous multiple vesicles
Català, 2022	Pfizer	Cross-sectional study	6.7 (163)			NR					
Vaccaro, 2022	Pfizer (n=12) and AstraZeneca (n=16)	Case series	28.6 (28)	4 and 12 hours	7-14	NR				Topical corticosteroids<br>Antihistamines	-Red-brown plaque with vesicular lesions
Rerknimitr, 2022	AstraZeneca	Prospective cohort study	2.94 (34)	10	3	NR					-Number of patients receiving first and second doses not specified<br>-N values represent the number of people who had reactions to each dose
Pourani, 2022	AstraZeneca	Cross-sectional Questionnaire	0.9% (332)			NR					-Number of patients receiving first and second doses not specified
Català, 2022	AstraZeneca	Cross-sectional study	8.4 (95)			NR					
Rerknimitr, 2022	Sinovac	Prospective cohort study	2.47 (162)	3*	7*	NR	2.11 (95)	8 hours*	8*		-Number of patients receiving first and second doses not specified<br>-N values represent the number of people who had reactions to each dose
Adya, 2021	AstraZeneca	Case report	100 (1)	4	3	No					-Papulovesicular lesions
Bullae or Bullous-like Reactions											

Freeman, 2022	Moderna	Registry Study	1.6 (427)			NR	0.5 (214)				
Larson, 2022	Moderna	Case series	8 (12)	14		No				Oral prednisone<br>Topical corticosteroids	
Tomayko, 2021	Moderna	Case series	50 (4)	8.5	1: Ongoing at 2 months<br>1: Resolved at 5 days	NR	50 (4)	12	Ongoing at 23 or 105		
Damiani, 2021	Moderna	Case series	100 (3)	6.7		Yes	33 (3)			Oral prednisone<br>Mycophenolate mofetil<br>Oral prednisone	-One case received the second vaccine after 28 days and had worsening of the bullous pemphigoid lesions<br>-Two cases received the second dose after 28 days with no significant symptoms
Khalid, 2021	Moderna	Case report	100 (1)	10	Ongoing	Yes	100 (1)	4		Topical corticosteroids	-Bullous pemphigoid likely diagnosis
Kong, 2021	Moderna	Case report				No	100 (1)	1	12+	Oral prednisone<br>Bullae drain<br>Mupirocin ointment with petroleum	
Freeman, 2022	Pfizer	Registry Study	4.4 (114)			NR	6.4 (140)				
Larson, 2022	Pfizer	Case series	8 (12)	21		No				Prednisone<br>Topical corticosteroids<br>Doxycycline<br>Niacinamide<br>Antihistamines	
Tomayko, 2021	Pfizer	Case series	37.5 (8)	5.7	1: Ongoing at 3 weeks<br>2: Resolved at 3 or 8 weeks	NR	62.5 (8)	6.8	3: Improving between 2-4 weeks<br>1: Resolved at 2 weeks<br>1: Ongoing at 6 weeks		
Juay, 2021	Pfizer	Case series	33 (3)	14		No	66 (3)	3		Dyshidrotic eczema treated with topical betamethasone dipropionate ointment; acute generalized exanthematous pustulosis treated with topical mometasone cream; bullous pemphigoid treated with prednisolone and clobetasol	-Vesiculobullous<br>-All cases resolved with treatment

Damiani, 2021	Pfizer	Case series	100 (2)	3		No				Oral prednisone	-Second dose vaccines given after 21 days with no significant symptoms
Coto-Segura, 2022	Pfizer	Case series	100 (4)	3 - 17		NR					-Number of patients receiving first and second doses not specified<br>-Morphology of lesions: urticated and erythematous plaques and tense bullae on an erythematous base, located on the trunk, forearms, and wrist. Small vesiculobullous, rosette-like pattern, on the thighs
Gambichler, 2022	Pfizer	Case series	100 (2)	4.5		Yes	100 (1)			Tapered systemic prednisolone	-Bullous pemphigoid
Dell'Antonia, 2022	Pfizer	Case report	100 (1)	7	Unresolved	Yes	100 (1)	3	21	Topical steroids<br>Prednisone with tapering	-Reaction recurred: new blisters on trunk and limbs, initially only on his legs
Young, 2022	Pfizer	Case report	100 (1)	3	Unresolved	Yes	100 (1)		90	Clobetasol propionate ointment<br>Emollient cream	
Pauluzzi, 2022	Pfizer	Case report	100 (1)	15	Ongoing at 7 weeks	NR			28	Intramuscular methylprednisolone acetate<br>Oral azathioprine	
Pérez-López, 2021	Pfizer	Case report	100 (1)	3	14	Yes	100 (1)			Topical corticosteroids<br>Prednisone	-78-year-old female with bullous pemphigoid
Nakamura, 2021	Pfizer	Case report	0 (1)			NR	100 (1)	3		Oral prednisolone<br>Steroid pulse therapy<br>IV immunoglobulin therapy	-83-year-old female with Bullous pemphigoid<br>-Oral prednisolone 30 mg/day tried first but was not effective; dosage was increased to 60 mg/day; then steroid pulse therapy and high-dose IV immunoglobulin therapy which were effective
Vaccaro, 2022	Pfizer (12)<br>AstraZeneca (16)	Case series	7 (28)	4 and 12 hours after both vaccines	7-14	NR				Topical corticosteroids<br>Antihistamines	
Agharbi, 2022	AstraZeneca	Case report	100 (1)	1		NR				Topical clobetasol propionate<br>Doxycycline	
Hali, 2022	AstraZeneca	Case report				NR	100 (1)	3	22+	Oral prednisolone	
Wantavornprasert, 2022	AstraZeneca	Case report	100 (1)	1	14	NR				Topical dexamethasone cream	

*Median
IV: Intravenous
IM: Intramuscular
NR: Not reported

Supplementary Table 3. New-Onset or Reactivation of Cutaneous Conditions After Vaccinations

Study (Author, year)	Vaccine Type	Type of study	% Of cases after 1st dose (n= total sample size for the vaccine type)	Time to onset after vaccine 1 (mean days)	Duration of reaction (mean days)	Did reaction recur with second dose	% Of cases after 2nd dose (n= total sample size for the vaccine type)	Time to onset after vaccine 2 (mean days)	Duration of reaction (mean days)	Treatment (if noted)	Additional comments
Herpes Zoster Activation											
Freeman, 2022	Pfizer	Registry Study	0.8 (114)			NR					
van Dam, 2021	Pfizer (Comirnaty; tozinameran)	Case series	100 (2)	14	10-14	No				Valacyclovir	
Mehta, 2022	AstraZeneca	Case series	33.3 (3)	3	14	NR				Valacyclovir	
Rerknimitr, 2022	AstraZeneca	Prospective cohort study	2.94 (34)	1	14	NR	9.09 (11)	3	12		-Unclear whether the herpes infection is a primary or reactivation<br>-Number of patients receiving first and second doses not specified<br>-N values represent the number of people who had reactions to each dose
Rerknimitr, 2022	Sinovac	Prospective cohort study	0.62 (162)	3	3	NR					-Unclear whether the herpes infection is a new-onset of reactivation<br>-Number of patients receiving first and second doses not specified<br>-N values represent the number of people who had reactions to each dose
Agarwal, 2022	AstraZeneca	Case series (n=16)	25 (4)	2		NR					-52-year-old male presenting with multiple grouped vesicles of herpes zoster
Herpes Zoster Reactivation											
Atiyat, 2021	Pfizer	Case report				NR	100 (1)	3	12	Admitted to ICU for alcohol withdrawal<br>Acyclovir<br>Cefazolin<br>Gabapentin<br>Bictegravir<br>Emtricitabine<br>Tenofovir	-36-year-old male, HIV positive presenting to the emergency department. He was admitted for alcohol withdrawal
Nanova, 2021	Pfizer	Case report	100 (1)	7		NR					-33-year-old female presenting to the emergency department with recurrence of varicella
Santovito, 2021	Pfizer	Case report				NR	100 (1)	3	30	Prednisone<br>Hydroxyzine<br>Mupirocin ointment with no improvement<br>Lesions self-regressed later	-27-year-old healthy male<br>-Papulovesicular rash consistent with VZV
Fukuoka, 2021	Pfizer		40 (5)	17.5		No	60 (5)	16		Four patients didn't receive treatment<br>One patient treated with antiviral drug due to involvement of their face	-Age range of 59-97, prior vaccination status unknown

Shah, S. 2021	Sinopharm (Vero cell)	Case report	100 (1) - didn't specify which dose or if he took 2 doses	5	12	NR				Valacyclovir<br>Mupirocin ointment	-51-year-old male
Aksu, 2021		Case report		9.8	6.75	NR	100 (1)	5		Valaciclovir<br>Acyclovir cream<br>Paracetamol	-68-year-old male presenting to the family medicine clinic with vesicular lesions
Psoriasis Reactivation											
Wei, 2022	Moderna	Case series	14 (7)	7		Yes	86 (7)	41		4: Improved with clobetasol, risankizumab, mometasone, triamcinolone, tildrakizumab, apremilast, or phototherapy<br>1: Resolved with apremilast, or tildrakizumab<br>1: Unknown status	-6 out of the 7 patients had a previous history of psoriasis.<br>-6 patients received the Moderna vaccine, while 1 received the Pfizer vaccine.<br>-One patient reported a flare in psoriasis after the first dose of the vaccine and a second exacerbation after the second dose.<br>-All the other patients only experienced symptoms after the second dose.<br>-The median latency for the onset of flare or new-onset psoriasis was 24 days following the administration of the second vaccine dose
Wei, 2022	Pfizer (n=38)<br>Moderna (n=34)<br>Janssen Pharmaceuticals (n=7)	Retrospective review	72 (79)	Moderna and Pfizer: 0-65<br>Janssen: 4-17		Yes					-Retrospective review of the CDC VAERS of all reports from December 2020 to August 2021.<br>-Majority received the Pfizer (48.1%), then Moderna (43.0%), then Janssen (8.9%) vaccine<br>-79 patients had new-onset or exacerbation of psoriasis<br>-57 had known psoriasis; 22 reported new-onset psoriasis<br>-Of the 57 patients whose symptoms began after the first dose of the Pfizer or Moderna, 5 patients reported worsening after the second dose; 14 reported onsets of symptoms after the second doses only.
Niebel, 2021	Pfizer	Retrospective analysis/case series				NR	7.7 (13)	20		Cignoline<br>Topical corticosteroids<br>Phototherapy<br>Tildrakizumab	-This retrospective analysis included 19 patients.
Piccolo, 2022	Pfizer	Case series	100 (2)	30		NR				Oral acitretin	-Average age of patients was 60 years.<br>-This case series describes patients with past history of plaque-type psoriasis presenting with vaccine-induced pustular psoriasis
Ricardo, 2021	Pfizer	Case report				NR	100 (1)	7	~105 days	Topical clobetasol ointment	-76-year-old female presenting with de novo nail psoriasis
Flare of immune complex vasculitis											
Niebel, 2021	Pfizer	Retrospective analysis/case series				NR	7.7 (13)	2		Dapsone<br>Prednisolone	

CDC: Centre for Disease Control and Prevention
VAERS: The Vaccine Adverse Event Reporting System
NR: Not Reported

Supplementary Table 4. Studies reporting common cutaneous manifestations of COVID-19 vaccines

Study (Author, year)	Vaccine Type	Type of study	% Of cases after 1st dose (n= total sample size for the vaccine type)	Time to onset after vaccine 1 (mean days)	Duration of reaction (mean days)	Did reaction recur with second dose	% Of cases after 2nd dose (n= total sample size for the vaccine type)	Time to onset after vaccine 2 (mean days)	Duration of reaction (mean days)	Treatment (if noted)	Additional Comments
Delayed Large Local Arm Reaction											
McMahon, 2021	Moderna	Randomized controlled trial	66 (267)	7*	4	NR	30 (102)	2*	3		
Freeman, 2022	Moderna	Registry Study	53.8 (427)			NR	23.4 (214)				-Registry study on skin reactions from COVID vaccine type by the American Academy of Dermatology/International League of Dermatological Societies<br>-As of August 16, 2021, the registry had 2063 cutaneous vaccine reactions from 870 patients
McMahon, 2021	Pfizer	Randomized controlled trial	15 (34)	7*	4	NR	18 (40)	2*	3		
Freeman, 2022	Pfizer	Registry Study	10.5 (114)			NR	12.1 (140)				
Niebel, 2021	Pfizer	Retrospective analysis/case series	7.1 (14)	1	~21	No				Prednisolone with tapering	-COVID arm was described as upper arm and shoulder erythematous and edematous, forearm swollen; erythema nodosum<br>-Mean age of the study was 48.9 years with 12 females and 7 males
Tammaro, 2021	Pfizer	Case series	100 (1)	7	~17	NR				Topical corticosteroid cream	-Severe xerosis and pruritus in the vaccine injection area<br>-Patient’s chest also had erythematous pruritic and painful rashes similar in onset and duration to what was found at the injection site
Rerknimitr, 2022	Sinovac	Prospective cohort study				NR	Delayed = 1.05 (95)	9*	5*		-Number of patients receiving first and second doses not specified<br>-N values represent the number of people who had reactions to each dose<br>-A delayed reaction was greater than or equal to 8 days later
Local Injection Site Reactions											
Baden, 2021	Moderna	Randomized	84 (14538)	<28		NR	88.8 (14538)	<28			

		controlled trial									
McMahon, 2021	Moderna	Randomized controlled trial	54 (267)	1*	4	NR	70 (102)	1*	3		
Parés-Badell, 2021	Moderna	Cross-sectional study	23.7 (506)		3*	NR	45.5 (121)		3*		-The patients had swelling and redness at the injection site<br>-80% were females and 77% were between ages 18-55<br>-Median age was 34 years
Valera-Rubio, 2022	Moderna	Prospective survey Study	51.75 (swelling) 41.74 (erythema) (599)	Within 7 days of vaccination		NR	45.9 (swelling) 37.9 (erythema) (595)	Within 7 days of vaccination		Analgesics Antipyretics	-Demographics: 79.1% female, 13% were 18-30 years old, 59.7% were 31-55 years old, and 27.3% were over 55 years old.<br>-Median age was 47 years
Larson, 2022	Moderna	Case series	8 (12)	7 days		No					
Sidlow, 2021	Moderna	Case series	33 (6)	1 w	3	Yes				Topical corticosteroids Self-limiting	-One case had an urticarial reaction recur second dose; the patient initially reacted after the first dose
Català, 2022	Moderna	Cross-sectional study	61.9 (147)	4.9	7.4	NR					
McMahon, 2021	Pfizer	Randomized controlled trial	24 (34)	1*	4	NR	25 (40)	1*	3		
Parés-Badell, 2021	Pfizer	Cross-sectional study	8.1 (2373)		3*	NR	13.4 (2344)		3*		-Reaction described as pain, redness, and swelling at the injection site
Valera-Rubio, 2022	Pfizer	Prospective Survey Study	27.38 (swelling) 14.15 (erythema) (650)	Within 7 days of vaccination		NR	24.77 (swelling) 14.62 (erythema) (639)	Within 7 days of vaccination		Analgesia Antipyretics	-Allergies correlated with a higher frequency of nausea (28% vs. 19.1%), redness (58.2% vs. 45.8%), swelling (66.4% vs. 53.7%) and itching (39.2% vs. 28.6%) at the injection site for Moderna compared to Pfizer<br>-Allergies correlated with a higher frequency of discomfort (64.1% vs. 54.3%), fatigue (66.4% vs. 57.4%), headache (50.2% vs. 40.5%), nausea (16.6% vs. 9.6%), injection site swelling (43% vs. 27.4%), and difficulty moving the arm (63.7% vs. 49.9%) for Pfizer compared to Moderna<br>-Comorbidities were associated with increased diarrhea (14.9% vs. 9.4%), redness (55.6% vs. 47%), injection site itching (38.7% vs. 28.5%), and anaphylaxis (4% vs. 1.4%) for Moderna compared to Pfizer

											-Comorbidities were associated with increased insomnia (15.1% vs. 9.4%) for Pfizer compared to Moderna
Ripabelli, 2022	Pfizer	Retrospective study	77.9 (340)	~ 4-24 hours	0.5-2	NR	65.6 (340)	~4-24 hours	~0.5 - 2	NSAIDs<br>Acetaminophen	-Reaction described as pain, redness, and swelling at the injection site<br>-Average age was 49.2 years
Cuschieri, 2021	Pfizer	Observational survey	87 (1480)			Yes	70.6 (1480)				-Reaction described as pain, redness, and swelling at the injection site<br>-66.7% female
Larson, 2022	Pfizer	Case series	8 (12)			No				Topical corticosteroids<br>Antihistamines	
Almufty, 2021	Pfizer	Cross-sectional study	66.9 (251)	< 24 h (38.8%)<br>1–3 days (54.6%)<br>4days – 1 week (5.1%)<br>> 1 week (1.5%)<br>(850 total subjects with adverse reactions to Pfizer, AstraZeneca, and Sinopharm)		NR					-This study did not differentiate between first and second doses, the different vaccine types, or the different reaction types<br>-Injection site reactions defined as pain and redness.
Català, 2022	Pfizer	Cross-sectional study	14.1 (163)			NR					

Vaccaro, 2022	Pfizer (12) and AstraZeneca (16)	Case series	53.6 (28)	4 and 12 hours after both vaccines	14 days	NR				Topical corticosteroids Antihistamines	-Information gathered from January 2nd, 2021, to March 31st, 2021<br>-16217 (9382 females [median age 33] and 6835 males [median age 53]) and 1377 (693 females and 683 males, median age 47.3) people received the Pfizer and AstraZeneca vaccines, respectively<br>-Reactions were described as pink or red-purple or red-brown<br>-The patches had a smooth surface and defined edges<br>-Most patients felt burning, itching, and pain on palpation
Rerknimitr, 2022	AstraZeneca	Prospective cohort study	Acute = 5.88 (34); delayed = 2.94 (34)	Acute= 13 hours* Delayed= 240 hours*	Acute:= 4.5* Delayed = 3*	NR	Acute = 9.09 (11)	5 hours	7		-Number of patients receiving first and second doses not specified<br>-N values represent the number of people who had reactions to each dose.<br>-An acute reaction was less than 8 days later, and a delayed reaction was greater than or equal to 8 days later
Almufty, 2021	AstraZeneca	Cross-sectional study	54.2 (533)	< 24 h (38.8%) 1–3 days (54.6%) 4 days – a week (5.1%) > 1 week (1.5%) (n= 850 total subjects with adverse reactions to Pfizer, AstraZeneca, and Sinopharm)		NR					
Català, 2022	AstraZeneca	Cross-sectional study	16.8 (95)			NR					

Rerknimitr, 2022	Sinovac	Prospective cohort study	Acute = 2.47 (162)	41.5 hours*	3*	NR	Delayed = 1.05 (95)	9*	5*		-Number of patients receiving first and second doses not specified<br>-N values represent the number of people who had reactions to each dose<br>-An acute reaction was less than 8 days later
Urticaria or urticarial reaction											
Baden, 2021	Moderna	Randomized controlled trial	0.2 (15185)	>28 days after first or second dose		NR					
McMahon, 2021	Moderna	Randomized controlled trial	5.9 (267)	3	5	NR	6.9 (102)	2	3		
Parés-Badell, 2021	Moderna	Cross-sectional study	3.8 (506)		3*	NR	5.0 (121)		3*		-Reaction included hives and rashes<br>-80% were females and 77% were between ages 18-55 years<br>-Median age was 34 years
Freeman, 2022	Moderna	Registry Study	6.6 (427)			NR	10.3 (214)				
Sidlow, 2021	Moderna	Case series	33 (6)	8		Yes	33 (6)	2	5		
Holmes, 2021	Moderna	Case series	33.3 (3)	2	3	Advised against 2nd dose due to risk of airway obstruction with asphyxiation/systemic circulatory symptoms				Oral diphenhydramine<br>Baking soda baths	-Average age was 54.4 years
Chopra, 2021	Moderna	Case report	100 (1)	9	30	Declined second vaccination				Prednisone with tapering<br>Triamcinolone cream	-56-year-old female with a dermal hypersensitivity reaction
Guénin, 2021	Moderna	Case report	100 (1)	8	10	Yes	100 (1)	2	2	Topical mometasone with minimal effect in reducing symptoms<br>Prophylactic loratadine prior to 2nd and 3rd doses	-61-year-old female
Myrdal, 2021	Moderna	Case report	100 (1)	9	2	No				Oral prednisone with tapering	-40-year-old male<br>-Reaction was pruritic, urticarial, indurated papules and plaques
Català, 2022	Moderna	Cross-sectional study	10.2 (147)	4.9	7.5	NR					

McMahon, 2021	Pfizer	Randomized controlled trial	26 (34)	3	5	NR	20.5 (40)	2	3		
Parés-Badell, 2021	Pfizer	Cross-sectional study	1 (2373)		3*	NR	1.9 (2344)		3*		-Reaction included hives and rashes<br>-80% were females and 77% were between ages 18-55 years<br>-Median age was 34 years
Freeman, 2022	Pfizer	Registry Study	21.1 (114)			NR	15 (140)				
Farinazzo 2021	Pfizer	Retrospective database review	17 (35)	3.9		No	2 (9)	1		IV steroids<br>Self-limiting	
Ripabelli, 2022	Pfizer	Retrospective study	1.5 (340)	~4-24 hours	~0.5-2	NR	0.6 (340)	~4-24 hours	~0.5-2	NSAIDs<br>Acetaminophen	
Niebel, 2021	Pfizer	Retrospective analysis/case series	7.1 (14)	2		No				Antihistamines	
Burlando, 2022	Pfizer (n=13)	Case series	20 (5)	10		NR					
Peigottu, 2021	Pfizer	Case series	66.7 (9)	Up to 1		Yes	33.3 (6)	30 minutes		Antihistamines<br>Steroids	-8 females, mean age 46 years<br>-Reactions were reported to the Italian Pharmacovigilance Authority
Holmes, 2021	Pfizer	Case series	0 (2)			NR	50 (2)	5	14	Oral prednisone<br>topical triamcinolone ointment<br>Oral fexofenadine<br>Oral diphenhydramine	-Reaction was described as urticarial vasculitis<br>-Average age was 54.4 years
Choi, 2021	Pfizer	Case series	75 (4)	~7 days	~28	Yes	33.3 (3)			Antihistamines	-Average age was 30.5 years<br>-One patient had recurrence after second dose
Català, 2022	Pfizer	Cross-sectional study	14.7 (163)			NR					
Rerknimitr, 2022	AstraZeneca	Prospective cohort study	11.76 (34)	72 hours*	4.5*	NR					-Number of patients receiving first and second doses not specified<br>-N values represent the number of people who had reactions to each dose<br>-The reaction was described as eczematous.
Rerknimitr, 2022	AstraZeneca	Prospective cohort study	26.47 (34)	21 hours*	4*	NR	27.27 (11)	4 hours*	3*		-Number of patients receiving first and second doses not specified<br>-N values represent the number of people who had reactions to each dose.
Niebel, 2021	AstraZeneca	Retrospective analysis/case series	33.3 (3)	1		NR				Antihistamines	-Mean age of the study was 48.9 years with 12 females and 7 males
Català, 2022	AstraZeneca	Cross-sectional study	21.1 (95)			NR					

Akdaş, 2021	Sinovac	Case series	50 (6)	28.1	Up to 84 days	Yes	60 (5)	2 hours	51.3	Oral antihistamine<br>Systemic corticosteroid<br>Omalizumab	-Average age of the case series was 37.5 years<br>-The Series involved healthcare workers in a single center<br>-One case only had a reaction to second dose<br>-One patient refused the second dose after reacting to the first dose<br>-Two cases who reacted after the first dose also had a recurrence reaction after the second dose
Agarwal, 2022	AstraZeneca (n=4)	Case series	50 (4)	1.5		NR					
Morbilliform or maculopapular eruption											
Baden, 2021	Moderna	Randomized controlled trial	<0.1 (15185)	>28 after first or second dose		NR					-Maculopapular eruption
McMahon, 2021	Moderna	Randomized controlled trial	4.1 (267)	3	4.5	NR	6.9 (102)	2	2.5		
Freeman, 2022	Moderna	Registry study	6.1 (427)			NR	7.9 (214)				
Burlando, 2022	Moderna (n=2)	Case series	100 (1)	3		NR					-Papular
Sidlow, 2021	Moderna	Case series	33 (6)	8	7 days	No					
Holmes, 2021	Moderna	Case series	0 (3)			NR	50 (2)	1	35	Prednisone with tapering<br>Cetirizine<br>Famotidine<br>Topical triamcinolone ointment<br>Oral fexofenadine<br>Hydroxyzine	-Morbilliform eruption<br>-Average age was 54.4 years
Català, 2022	Moderna	Cross-sectional study	4.1 (147)	4	10.3	NR					
McMahon, 2021	Pfizer	Randomized controlled trial	18 (34)	3	4.5	NR	7.5 (40)	2	2.5		
Freeman, 2022	Pfizer	Registry study	14 (114)			NR	10.7 (140)				
Annabi, 2021	Pfizer (n=5)	Retrospective observational study/case series	40 (5)	7.5	11.5	No				Topical corticosteroids	-Maculopapular drug reaction (morbilliform rash)<br>-Average age of the study was 61.5 years<br>-Retrospective observational study for patients referred to the Dermatology Department of Cochin Hospital from Jan 2021-Apr 2021

Burlando, 2022	Pfizer (n=13)	Case series	40 (5)	2		NR	12.5 (8)	4 hours			
Peigottu, 2021	Pfizer	Case series	11.1 (9)	1		Patient who reacted to the first dose did not receive second dose	66.7 (6)	1-2		Antihistamine<br>Steroids	
Català, 2022	Pfizer	Cross-sectional study	11.7 (163)			NR					
Rerknimitr, 2022	Astra Zeneca (n=2)	Prospective cohort study				NR	9.09 (11)	0.25	1		-Number of patients receiving first and second doses not specified<br>-N values represent the number of people who had reactions to each dose
Annabi, 2021	Astra Zeneca (n=2)	Retrospective observational study/case series	50 (2)	3	30	No				Topical corticosteroids	-Maculopapular pustular exanthema<br>-Average age was 61.5 years
Burlando, 2022	AstraZeneca (n=6)	Case series	33 (3)	1		NR					
Català, 2022	AstraZeneca	Cross-sectional study	11.6 (95)			NR					
Rerknimitr, 2022	Sinovac	Prospective cohort study	2.47 (162)	11 hours*	2.5	NR	1.05 (95)	7	7		-Number of patients receiving first and second doses not specified<br>-N values represent the number of people who had reactions to each dose
Akdaş, 2021	Sinovac	Case series	16.7 (6)	7	7	Yes	20 (5)	1	56	Systemic corticosteroid<br>Antihistamine<br>Topical corticosteroid	-Maculopapular<br>-Average age of the case series was 37.5 years<br>-The recurrence reactions after the second vaccine included atypical targetoid lesions, more extensive skin involvement, and erythematous patch
Pityriasis rosea-like eruption											
McMahon, 2021	Moderna	Randomized controlled trial	0.4 (267)	14	10	NR	0 (102)	4	5		
Freeman, 2022	Moderna	Registry Study	2.3 (427)			NR	2.8 (214)				
Larson, 2022	Moderna	Case series	8 (12)	7		No				Topical corticosteroid	

Català, 2022	Moderna	Cross-sectional study	3.4 (147)	6.3	25.2	NR					
McMahon, 2021	Pfizer	Randomized controlled trial	5.9 (34)	14	10	NR	2.5 (40)	4	5		
Freeman, 2022	Pfizer	Registry Study	2.6 (114)			NR	3.6 (140)				
Farinazzo 2021	Pfizer (n=46)	Retrospective database review (n=46)	2 (35)	5		No	11 (9)	4		Self-limiting	
Niebel, 2021	Pfizer	Retrospective analysis/case series				NR	7.7 (13)	8		Topical corticosteroids	
Burlando, 2022	Pfizer (n=13)	Case series				NR	12.5 (8)	30			
Choi, 2021	Pfizer	Case series	25 (4)	3		Did not receive second dose					-Papulosquamous eruption diagnosed as pityriasis rosea<br>-Average age was 30.5 years
Sernicola, 2022	Pfizer	Case report	100 (1)	10	~60	No	0 (1)			Methylprednisolone with tapering	-31-year-old female with pityriasis lichenoides et varioliformis acuta-like eruption
Hunjan, 2022	Pfizer	Case report	100 (1)	3		Yes	100 (1)	~3		Enstilar foam<br>Emollients<br>Acitretin<br>Topical mometasone	-51-year-old male; scaly, erythematous rash which worsened after second dose<br>-Patient ended up in the acute medical unit being mildly hypotensive and tachycardic<br>-Pityriasis rubra pilaris-like eruption
Català, 2022	Pfizer	Cross-sectional study	6.7 (163)			NR					
Niebel, 2021	AstraZeneca	Retrospective analysis/case series	33.3 (3)	22		Unsure (schedule for Pfizer 2nd dose but not received yet)				Topical corticosteroids<br>Emollient<br>Watchful waiting	
Mehta, 2022	AstraZeneca	Case series	33.3 (3)	1	Lost to follow up	NR				Topical mometasone<br>Oral paracetamol	-Average age was 34.7 years.

Leerunyakul, 2021	AstraZeneca	Case report	100 (1) - didn't specify first or second dose/how many doses patient took	14	7	NR				Topical triamcinolone	-52-year-old female
Català, 2022	AstraZeneca	Cross-sectional study	4.2 (95)			NR					
Rerknimitr, 2022	Sinovac	Prospective cohort study	0.62 (162)	1	14	NR	1.05 (95)	1	4		-Number of patients receiving first and second doses not specified<br>-N values represent the number of people who had reactions to each dose.
Akdaş, 2021	Sinovac	Case series	16.7 (6)	4	21	Yes	20 (5)	4	56	Topical corticosteroid	
Pernio/Chilblains											
McMahon, 2021	Moderna	Randomized controlled trial	1.1 (267)	10	10.5	NR	0 (102)				
Freeman, 2022	Moderna	Registry Study	1.6 (427)			NR	1.4 (214)				
Holmes, 2021	Moderna	Case series	33.3 (3)	10	13	No				Oral diphenhydramine topical hydrocortisone	-Chilblains-like dermal hypersensitivity reaction<br>-Average age of the series was 54.4 years
McMahon, 2021	Pfizer	Randomized controlled trial	8.8 (34)	10	10.5	NR	5 (40)	11	4.5		
Freeman, 2022	Pfizer	Registry Study	4.4 (114)			NR	5 (140)				
Farinazzo 2021	Pfizer	Retrospective database review	3 (35)	4		Yes				Self-limiting	
Annabi, 2021	Pfizer (n=5)	Retrospective observational study/case series	20 (5)	4	7	Yes	16.7 (6)	5	7	Self-limited	-One case had 2 different manifestations after the first and second doses: erythematous nodules after first dose and chilblains after second dose
Dermatitis											

Baden, 2021	Moderna	Randomized controlled trial	0.1 (15185)	>28 after first or second dose		NR					
McMahon, 2021	Moderna	Randomized controlled trial	1.1 (267)	5.5	4.5	NR	0 (102)	5	8		
Freeman, 2022	Moderna	Registry Study	1.2 (427)			NR	1.4 (214)				-Contact dermatitis
McMahon, 2021	Pfizer	Randomized controlled trial	1 (34)	5.5	4.5	NR	5 (40)	5	8		
Freeman, 2022	Pfizer	Registry Study	0 (114)			NR	3.6 (140)				-Contact dermatitis
Niebel, 2021	Pfizer	Retrospective analysis/case series	14.3 (14)	2.5		Yes	7.7 (13)	12	Up to 14+	Prednisolone<br>Cyclosporine<br>Topical corticosteroids	-The reactions after the second vaccine included hematogenous contact dermatitis and flare of chronic hand eczema
Niebel, 2021	Pfizer	Retrospective analysis/case series	7.1 (14)	21	~ 7	No				Prednisolone with tapering<br>Topical corticosteroids<br>Phototherapy	-Psoriasiform flare of atopic dermatitis
Annabi, 2021	Pfizer (n=5)	Retrospective observational study/case series	20 (5)	5		Yes	16.7 (6)	4		Topical corticosteroids<br>Phototherapy	
Holmes, 2021	Pfizer	Case series	50 (2)	7		Yes	50 (2)		14-21	Triamcinolone<br>Tacrolimus ointment<br>Fluocinonide ointment	-Average age was 54.4 years
Leasure, 2021	Pfizer	Case series	100 (2)	3.5	14+	Yes	100 (2)	9		Topical corticosteroids<br>Oral antihistamines<br>Oral prednisone with tapering	-Patients were a 43-year-old male and a 51-year-old female with a history of dyshidrotic eczema<br>-Patients had eczematous eruption/dermatitis after vaccines
Cutaneous small vessel vasculitis											
Sandhu, 2021	AstraZeneca	Case series	100 (2)	6	10.5	Yest	50 (2)	2		Oral prednisolone with tapering<br>Topical corticosteroids	-Average age was 51.5 years<br>-One patient had a recurrence of reaction after the second dose after initially reacting to the first dose
Shahrigharahkoshan, 2021	AstraZeneca	Case report	100 (1)	10		Recommended different vaccine for second dose with				Prednisolone with taper<br>Dapsone	-77-year-old female presenting to the dermatology center with maculopapular eruptions, erythematous plaques, and bullae

						no reaction					
Jin, 2022	AstraZeneca	Case report	100 (1)	7	~42	Yes	100 (1)		~3	Oral methylprednisolone with tapering<br>Colchicine<br>Topical methylprednisolone	-68-year-old female with erythematous to purpuric non-blanching macules
Fiorillo, 2022	AstraZeneca	Case report				NR	100 (1)	5	~14	Oral prednisone with tapering	
Rerknimitr, 2022	Sinovac	Prospective cohort study	0.62 (162)	4 hours	36	NR					-Number of patients receiving first and second doses not specified<br>-N values represent the number of people who had reactions to each dose
Kharkar, 2021	Covaxin	Case report				NR	100 (1)	4	10	Rest<br>Leg elevation<br>Antihistamines	-31-year-old female presenting with purpuric lesions on legs; cutaneous small vessel vasculitis with an asymmetrical distribution<br>-This vaccine is available in Bharat Biotech, Hyderabad, India
Berry, 2021	Janssen	Case report	100 (1)	7	~4-7	NR				Oral prednisone<br>Topical triamcinolone cream<br>Oral and IV analgesics	-65-year-old African American man presenting with tender petechiae and purpura on the lower portions of bilateral extremities, petechiae on the lower portion of the abdomen, and palpable purpura on the left arm. He was admitted to the hospital for further workup and management. Histopathologic results of skin biopsy revealed features consistent with leukocytoclastic vasculitis
Ecchymosis, Petechiae, Purpura											
Freeman, 2022	Moderna	Registry Study	0.2 (427)			NR	1.4 (214)				
Freeman, 2022	Pfizer	Registry Study	2.6 (114)			NR	0.7 (140)				
Rerknimitr, 2022	AstraZeneca	Prospective cohort study	8.82 (34)	2*	10*	NR					-Number of patients receiving first and second doses not specified<br>-N values represent the number of people who had reactions to each dose
Rerknimitr, 2022	Sinovac	Prospective cohort study	3.09 (162)	10 hours*	7*	NR	2.11 (95)	8 hours*	13.5*		-Number of patients receiving first and second doses not specified<br>-N values represent the number of people who had reactions to each dose
Lymphadenopathy											
Parés-Badell, 2021	Moderna	Cross-sectional study	3.8 (506)		3*	NR	9.9 (121)		3*		-Study was a cross-sectional study with an online ad hoc survey of healthcare workers (self-reported)<br>-80% were females and 77% were between ages 18 55 years

											-Median age was 34 years
Parés-Badell, 2021	Pfizer	Cross-sectional study	1.7 (2373)		3*	NR	6 (2344)		3*		
Ripabelli, 2022	Pfizer	Retrospective study	2.4 (340)	~4-24 hours	~2-3	NR	6.7 (340)	~4-24 hours	~2-3	NSAIDs Acetaminophen	

*Median
IV: Intravenous
IM: Intramuscular
NR: not reported
NSAIDs: Non-steroidal anti-inflammatory drugs

Supplementary Table 5. Cases of uncommon cutaneous manifestations of COVID-19 Vaccines

Study (Author, year)	Vaccine Type	First or Second Dose	Time to onset after vaccine (mean days)	Duration of reaction (mean days)	Did the reaction recur after the second dose	Treatment (if noted)	Additional Comments
Sweet's Syndrome							
Torrealba-Acosta, 2021	Moderna	First	3	~5	N/A	Methylprednisolone	-Morphology: deep red, non-scaly, edematous papules coalescing into plaques on the abdomen, upper chest, proximal upper extremities, bilateral upper flanks and back, with scattered non follicular pustules
Baffa, 2021	Pfizer	First	3	21	N/A	Methylprednisolone<br>Oral prednisone with tapering	
Majid, 2021	AstraZeneca	First	7	28	N/A	IM dexamethasone<br>Oral colchicine<br>Topical corticosteroid	-65-year-old female
Žagar, 2022	AstraZeneca	First	10		Patient was recommended not to receive second dose		
Radiation recall dermatitis**							
Afacan, 2021	Sinovac	First	5		N/A		-60-year-old female
Papular acrodermatitis							
Villagrasa-Boli, 2022	Pfizer	First and Second	3 after first;<br>13 after second	2-3	No	Topical betamethasone	-Lesions were symmetrical with erythematous papules and plaques affecting hands, elbows, and knees; coalescing erythematous papules and plaques on the back of the hands, elbows, knees, and feet
Acute generalized exanthematous pustulosis							
Agaronov, 2021	Moderna	First	Eruption spread after 3 days	12	NR	Diphenhydramine<br>Topical hydrocortisone cream	-27-year-old female<br>-There was no improvement with treatments offered
Toxic Epidermal Necrolysis							
Bakir, 2021	Pfizer	First	7	22	NR	Etanercept<br>IV fluid replacement<br>Nutrition support<br>Topical antibiotics<br>Ttopical lubricants	-49-year-old female presented to the emergency room and hospitalized
Stevens-Johnson syndrome							
Elboraey, 2021	Pfizer	Second	5		N/A	Oral prednisolone<br>Oral corticosteroids<br>Triamcinolone acetonide	-Middle-aged female patient presenting with oral lesions indicative of SJS
Dash, 2021	Unspecified	First	3	17	Deferred 2nd dose	Paracetamol<br>Levocetirizine<br>Oral Cyclosporine	-60-year-old male presenting with purpuric macules, bullae lesions
Necrosis							
Gruenstein, 2021	Pfizer	Second	7	75	N/A		-62-year-old male with HTN, diabetes mellitus, epilepsy, stage IV B-cell lymphoma, treated with rituximab

							-Presented with bullae and then necrosis at injection site reaction
Vitiligo							
Kaminetsky, 2021	Moderna	First	Several days later		Yes	Topical calcineurin inhibitor<br>Phototherapy	-61-year-old female. After the second dose, the vitiligo progressed/spread, and the patient presented to the clinic on day 3 after the vaccine
Cutaneous lichen planus (drug-induced)							
Merhy, 2021	Pfizer	First	7		NR		-56-year-old female
Erythema Multiforme							
Wunderlich, 2022	Pfizer	Second	2		N/A	Topical corticosteroids<br>Adhesive ointment containing corticosteroids for mucous membrane	-The lesions were maculopapular lesions with central blistering/crusting and an erythematous border. Sharply defined efflorescence on the elbow. Pseudomembranous ulcers on lips
T-cell–predominant cutaneous lymphoid hyperplasia							
LeWitt, 2022	Pfizer	Second	~3				
Cutaneous lupus erythematosus							
Niebel, 2021	Moderna	First and Second	10	~21	Yes	Prednisolone with tapering<br>Etoricoxib<br>Antihistamines<br>Topical corticosteroids	
Niebel, 2021	Pfizer	First	10	~21	No	Prednisolone (failed)<br>Pulse therapy with prednisolone with tapering<br>Topical mometasone furoate ointment	-73-year-old female with subacute cutaneous lupus erythematosus who developed disease exacerbation<br>-Patient was in full remission since Jan 2020
Niebel, 2021	Pfizer	First	7.5	~21	No	Prednisolone with tapering<br>Hydroxychloroquine<br>Methotrexate	-2 cases, one of which was a flare of cutaneous lupus erythematosus
Kreuter, 2022	Pfizer	First	10	~28	NR	Hydroxychloroquine<br>IV glucocorticosteroid with tapering	-79-year-old male<br>-Vaccine-induced eruption of cutaneous lupus erythematosus -Morphology: annular papulosquamous plaques and macules
Lichenoid Reactions							
Onn, 2021	Pfizer	First	12		Did not receive second dose	Patient was hospitalized<br>Cetirizine<br>Topical steroid<br>Oral prednisone<br>Oral famotidine<br>IV hydrocortisone<br>Phototherapy as an outpatient	-53-year-old healthcare worker presenting to the dermatology clinic and emergency department<br>-The lesions were papular erythematous skin eruptions and systemic reaction
Ziraldo, 2021	AstaZeneca	First	3 weeks	~13	NR	Degressive general corticosteroid therapy	-Drug-induced lichenoid exanthema<br>-Patch tests were negative for each component of AstraZeneca.
Pityriasis Lichenoides et Varioliformis Acuta (PLEVA)							

LeWitt, 2022	Pfizer	First and Second	2 weeks after first<br>1 week after second	~90	Yes	Doxycycline	
Molluscum contagiosum							
Piccolo, 2022	Pfizer	Second	A couple of days later		Patient refused second dose of vaccine	No treatment	-63-year-old female presenting with vaccine-induced reaction around molluscum contagiosum with secondary partial clearance of lesions
Cutaneous mucormycosis							
Shah, KM. 2021	Moderna	First	2	14	No	IV micafungin	-94-year-old male with a history of bullous pemphigoid presenting with cutaneous mucormycosis
Livedo racemosa							
Annabi, 2021	Pfizer	First	12	60	No	None	
Edematous infiltrated plaques							
Annabi, 2021	AstraZeneca	First	3	16	No	None	-44-year-old male with fever and edematous infiltrated plaques on buttocks, pathology showed papillary dermal edema with perivascular lymphocytic infiltrate.
Fixed drug eruption							
Annabi, 2021	Moderna	Second	2	5	N/A	Topical corticosteroids	
Niebel, 2021	Pfizer	First	10	~42	No	Prednisolone	-30-year-old female presenting with neutrophilic drug eruption, non-scaling erythematous plaques with neutrophils
Erythema nodosum							
Herrera, 2021	Moderna	Second	7		N/A	NSAIDs	-44-year-old female presenting to the emergency department
Mehta, 2022	AstraZeneca	First	7	17	No	Topical mometasone cream<br>Oral paracetamol	
Evan's Syndrome							
Hidaka, 2022	Pfizer	First and Second			NR	Prednisolone	-53-year-old female<br>-After first vaccine, patient had purpura in extremities and wheezing for a few days)<br>-After second vaccine, patient had thrombocytopenia and mild anemia
Vulvar Aphthous							
Popatia, 2022	Pfizer	Second	2	10	N/A	Lidocaine<br>Triamcinolone ointment<br>Acetaminophen<br>Ibuprofen	-Febrile female presenting with edema of the right labium majus and minus with three, tender,<br>-0.8–1 cm ulcers with a red rim, white-gray, necrotic crust
Angioedema							
Freeman, 2022	Moderna	First and Second			Yes		
Freeman, 2022	Pfizer	First and Second			Yes		
Rerknimitr, 2022	Sinovac	First	1*	1*	NR		
Dermatomyositis							
Niebel, 2021	Pfizer	First	30	~42	No	Prednisolone with tapering	
Psoriasis (New-Onset)							

Wei, 2022	Moderna	Second	24		N/A	Ixekizumab<br>Acitretin	-All 7 patients experienced new-onset or flares in psoriasis<br>-6 patients received the Moderna vaccine<br>-1 received the Pfizer vaccine<br>-1 patient reported a flare in psoriasis after the first dose of the vaccine and a second exacerbation after the second dose<br>-6 patients only experienced symptoms after the second dose
Wei, 2022	Pfizer (n=38)<br>Moderna (n=34)<br>Janssen Pharmaceuticals (n=7)	First		0-65 for Moderna and Pfizer<br>4-17 for Janssen	NR		-Retrospective review of the CDC VAERS of all reports from December 2020 to August 2021<br><br>-79 patients with new onset or exacerbation of psoriasis. 57 had known psoriasis, and 22 reported new-onset psoriasis. -Majority received the Pfizer vaccine (48.1%), then Moderna (43.0%), and then Janssen (8.9%) vaccine
New-Onset Dermatologic Conditions							
Freeman, 2022	Moderna	First and Second			NR		-First dose: lichen planus (4); psoriasis (1); possible leukocytoclastic vasculitis (1); Acne vulgaris (1)<br>-Second dose: granuloma annulare (1); lichen planus (1); psoriasis (1)
Freeman, 2022	Pfizer	Second			N/A		-Second dose: lichen planus (2), granuloma annulare (1), morphea (1), Raynaud (1), pityriasis lichenoides (1); lichen striatus versus inflammatory linear verrucous epidermal nevus versus Wolf isotopic response (1); unspecified toe rash (1)
Dermal Filler Reactions							
Osmond, 2021	Moderna	Second	1	2	N/A	Self-limiting	-Patient received Juvéderm Volux injection to the chin and jaw 3 years prior<br>-Chin enlargement after 24 h. Slurred speech, paresthesia of the lower face, headache, and malaise<br>-Likely delayed-type hypersensitivity
Michon, 2021	Pfizer	First	2	Case 1: 5<br>Case 2: 3.5 weeks	NR	Case 1: Self-limiting<br>Case 2: filler product dissolved with 75 units of hyaluronidase at a concentration of 150 units/ml.	-Case 1: Presenting with left tear trough swelling, erythematous, and tender<br>Patient received Juvéderm Volite to tear trough 1 year prior<br><br>-Case 2: Presenting with intermittent facial swelling primarily at the cheeks and under the eye. Swelling worse at 72 hours. follow up. Soft, mildly tender definite swelling
Savva, 2021	Pfizer	First and Second	2 after first<br>60 after second	7 (first dose)<br>5 (second dose)	Yes	Self-limiting after first dose<br>Methylprednisolone after second dose	-Patient received hyaluronic acid injections 1 year prior on the vermillion border of upper and lower lips<br>-Morphology first dose: small erythematous nodules on upper and lower<br>-Second dose: mild tenderness on her upper lip and erythematous edema

*Median
**RRD is characterized by erythema, edema, urticaria-like lesions, desquamation, vesiculation and, in severe cases, necrosis and ulceration
IV: Intravenous
IM: Intramuscular
NR: Not Reported
N/A: Not applicable
NSAIDs: Non-steroidal anti-inflammatory drugs
CDC: Centre for Disease Control and Prevention

VAERS: The Vaccine Adverse Event Reporting System
